# A Real-World Systematic Analysis of Driver Mutations’ Prevalence in Early- and Advanced-Stage NSCLC: Implications for Targeted Therapies in the Adjuvant Setting

**DOI:** 10.3390/cancers14122971

**Published:** 2022-06-16

**Authors:** Irene Terrenato, Cristiana Ercolani, Anna Di Benedetto, Enzo Gallo, Elisa Melucci, Beatrice Casini, Francesca Rollo, Aldo Palange, Paolo Visca, Edoardo Pescarmona, Enrico Melis, Filippo Gallina, Andrea Sacconi, Fabiana Letizia Cecere, Lorenza Landi, Federico Cappuzzo, Gennaro Ciliberto, Simonetta Buglioni

**Affiliations:** 1 UOSD Clinical Trial Center, Biostatistics and Bioinformatics, IRCCS Regina Elena National Cancer Institute, 00144 Rome, Italy; irene.terrenato@ifo.it (I.T.); andrea.sacconi@ifo.it (A.S.); 2 Pathology Unit, IRCCS Regina Elena National Cancer Institute, 00144 Rome, Italy; cristiana.ercolani@ifo.it (C.E.); anna.dibenedetto@ifo.it (A.D.B.); enzo.gallo@ifo.it (E.G.); elisa.melucci@ifo.it (E.M.); beatrice.casini@ifo.it (B.C.); francesca.rollo@ifo.it (F.R.); aldo.palange@ifo.it (A.P.); paolo.visca@ifo.it (P.V.); edoardo.pescarmona@ifo.it (E.P.); 3 Thoracic Surgery, IRCCS Regina Elena National Cancer Institute, 00144 Rome, Italy; enrico.melis@ifo.it (E.M.); filippo.gallina@ifo.it (F.G.); 4 Division of Medical Oncology 1, IRCCS Regina Elena National Cancer Institute, 00144 Rome, Italy; fabiana.cecere@ifo.it; 5 Division of Medical Oncology 2, IRCCS Regina Elena National Cancer Institute, 00144 Rome, Italy; lorenza.landi@ifo.it (L.L.); federico.cappuzzo@ifo.it (F.C.); 6 Scientific Direction, IRCCS Regina Elena National Cancer Institute, 00144 Rome, Italy; gennaro.ciliberto@ifo.it

**Keywords:** NSCLC, lung cancer targeted therapies, *EGFR*-mutated lung cancer, actionable mutations in NSCLC, early-stage NSCLC

## Abstract

**Simple Summary:**

The development of oncogene-targeted drugs has radically changed the course of non small cell lung carcinoma (NSCLC) in the advanced stage. Recently, the ADAURA trial demonstrated the efficacy of Osimertinib also in the adjuvant setting of *EGFR*-mutated NSCLC. This raises the question regarding whether the same paradigm applies also to currently approved drugs directed against non-*EGFR* NSCLC drivers. Herein we compared actionable genomic alterations in early- and advanced-stage NSCLC in 1961 unselected single-institution cases analyzed by routine molecular diagnostics procedures. Our data add significantly to the currently limited real-world data on actionable mutations in surgically resectable NSCLC. Our finding that distinct NSCLC genomic drivers are mutated at similar frequencies in early- and advanced-stage tumors implies that the relative biological potency of currently actionable NSCLC genomic drivers is conserved throughout clinical evolution and supports the hypothesis that genotype-matched therapies are likely to provide significant benefit in an adjuvant setting

**Abstract:**

The approval of osimertinib for adjuvant treatment of stage I–II–III *EGFR*-mutated NSCLC (early stage) represents a paradigm shift, raising the question of whether other genotype-matched therapeutics approved for advanced-stage NSCLC can also provide clinical benefit in the adjuvant setting. However, there is a paucity of real-world data on the prevalence of actionable genomic alterations (GAs) in early-stage NSCLC. We used next-generation sequencing, complemented by immunohistochemistry and fluorescence in situ hybridization, to screen our single-institution cohort of 1961 NSCLC consecutive cases for actionable molecular targets. The prevalence of actionable GAs was comparable in early versus advanced-stage NSCLC, the only exception being *KRAS* mutations (more frequent in early-stage cases). Consistent with advanced-stage tumors being more aggressive, co-occurrence of *TP53* and *EGFR* GAs as well as copy number gains were less frequent in early-stage tumors. *EGFR* mutations and high expression of PD-L1 were inversely associated, whereas *KRAS* mutations and high PD-L1 reactivity showed positive association. Recapitulating advanced-stage tumors, early-stage NSCLC had the highest share of *EGFR* mutations in lepidic and acinar subtypes. Resected lepidic tumors contained the highest proportion of the *KRAS* G12C actionable variant. These results, obtained with routine diagnostic technologies in an unselected clinical setting, provide a significant addition of real-world data in early-stage NSCLC.

## 1. Introduction

Lung cancer is one of the most common malignant tumors, with an incidence worldwide second only to non-melanoma skin cancer [1]. Lung cancer is also the leading cause of tumor-related deaths and disability-adjusted life years in males and the second most common cause in females [2]. Non-small-cell lung cancer (NSCLC) is the most common subtype of lung cancer, accounting for about 85% of cases [3,4]. Although a majority of patients with NSCLC are diagnosed with advanced, unresectable disease, surgery is the primary treatment in about 30% of cases, and especially those with early (stage I–IIIA) disease at diagnosis [5,6]. Because surgery alone may not be curative, adjuvant platinum-based chemotherapy is recommended for stage II to IIIA patients and in selected stage IB patients [2]. However, the risk of disease recurrence and disease-related death for these patients remains high. In fact, adjuvant chemotherapy is associated with only an 11% reduction in the risk of death at 5 years for stage II–IIIA resected NSCLC [7,8,9,10,11].

Within NSCLC, lung adenocarcinoma (LUAD) has witnessed major advances in therapeutic options for patients with locally/advanced metastatic disease, thanks to the advent of genotype-matched therapies [12]. Consequently, genomic analyses have become standard routine procedure for identifying therapeutically actionable mutations in advanced-stage LUAD patients. Recent results on the therapeutic targeting of *EGFR* by osimertinib suggest that targeted therapies may soon enter the arena of adjuvant LUAD treatment as well.

Osimertinib is an orally available *EGFR* tyrosine kinase inhibitor (TKI) approved as standard of care for *EGFR*-mutated advanced NSCLC [13,14,15]. The ADAURA double-blind, randomized phase 3 trial was designed to test the efficacy and safety of osimertinib versus placebo as adjuvant treatment in *EGFR*-mutated NSCLC. Interim analysis of the ADAURA trial at the end of the third year (i.e., two years earlier than the planned trial termination) revealed that osimertinib reduced the overall risk of disease recurrence or death by 83%; concerning CNS disease recurrence or death, the risk in the osimertinib group was reduced by 82% [16]. Overall survival data were not mature at the time of the published interim analysis. Based on these results, in December 2020 the FDA approved osimertinib for adjuvant treatment of surgically resected stage IB–IIIA NSCLC with deletions in exon 19 (Ex19del) or the exon 21 p.L858R mutation in *EGFR*. Following the FDA decision [17], in April 2020 the EMA issued a similar recommendation for adjuvant osimertinib monotherapy in eligible *EGFR*-mutated NSCLC.

The above results represent an unprecedented step forward in the adjuvant treatment of NSCLC and raise the question whether improved biomarker-based stratification could further improve the clinical management of mutated NSCLC in the adjuvant setting. Specifically, results from the ADAURA trial suggests that other genotype-matched therapeutics currently approved for advanced NSCLC patients may also provide clinical benefit in the adjuvant setting. In this regard, studies led by large international consortia, such as The Cancer Genome Atlas, indicated that the prevalence of currently actionable driver mutations was similar when comparing early-stage versus advanced NSCLC. It must be noted though that clinical genomics studies have so far solidified NGS analyses primarily in the advanced setting, the reason being that genotype-matched therapeutics received regulatory approval only for advanced NSCLC patients. As a consequence, there is a paucity of data comparing the bio-molecular profile of early- versus advanced-stage NSCLC in the routine clinical setting, i.e., in large series of unselected patients diagnosed with routine targeted NGS analyses in lieu of whole exome or whole genome sequencing platforms adopted by research-oriented international consortia.

In the present study, we compared the prevalence of actionable genomic vulnerabilities between early- and advanced-stage NSCLC—with a more detailed focus on the *EGFR* mutational landscape—in a series of 1961 consecutive LUAD patients referred to the Regina Elena National Cancer Institute between 2016 and 2019. Considering the increasing appreciation of the role played by tumor-cell intrinsic cues in shaping the immunobiology of the tumor microenvironment [18], we extended our analyses to PD-L1 expression, i.e., the biomarker used to assign NSCLC patients to immune checkpoint blockade-based therapies.

## 2. Materials and Methods

### 2.1. Patients and Tissue Specimens

From a total sample of 1961 NSCLC cases with adenocarcinoma histology, we identified 513 early-stage patients who had been surgically treated in our Institute from January 2016 to December 2019. All patients were of Caucasian descent. Pathologic diagnosis and staging were performed according to the current World Health Organization classification and TNM staging system of the International Association for the Study of Lung Cancer [4]. All clinical data were obtained both from inpatient and outpatient medical records. Defined patients who met the following inclusion criteria were enrolled: written informed consent; age ≥ 18 years; histologically confirmed NSCLC; sufficient FFPE tissue available for molecular analysis; demographic data, including age, sex, histologic type, and disease stage; did not receive preoperative systemic or radiation therapy. All formalin-fixed paraffin-embedded (FFPE) tissue sections were reviewed by pathologists for confirmation of histology and assessment of tumor content.

### 2.2. Immunohistochemistry (IHC)

A total of 1961 NSCLC samples, collected from January 2016 to March 2021, underwent immunohistochemical analyses for ALK, and ROS1 and 1351 of these were also analyzed for PD-L1 expression. The number regarding PD-L1 is lower for two main reasons: (1) the immunohistochemical test had been carried out previously on outpatients in other institutions; (2) the samples were not eligible for PD-L1 immunohistochemical analysis.

Three-μm sections of FFPE tumor samples were cut on SuperFrost Plus slides (Menzel-Gläser, Braunschweig, Germany).

VENTANA anti-ALK (D5F3) rabbit monoclonal primary antibody CDx Assay (CE IVD) stained with the OptiView DAB Detection and Amplification Kit was used for qualitative detection of ALK protein expression on a BenchMark ULTRA automated staining instrument (Ventana Medical Systems, Inc., Tucson, AZ, USA).

ROS1 immunoreactions were revealed by Bond Polymer Refine Detection on an automated autostainer (BondTM Max, Leica Biosystems, Milan, Italy) by using ROS1 (D4D6) rabbit monoclonal antibody (Cell Signaling Technology, Danvers, MA, USA). Evaluation of the ALK and ROS1 IHC results were performed independently and in a blinded manner by two investigators following, for ALK, the VENTANA Interpretation Guide. ROS1 results were considered negative and positive with an immune score of 0 and 3+, respectively. An IHC score 1+/2+ was considered equivocal and required a reflex fluorescent in situ hybridization (FISH) test for confirmation.

PD-L1 immunoreactions were revealed by using the EnVision FLEX visualization system on an Autostainer Link 48 (Agilent, Santa Clara, CA, USA) with PD-L1 pharmDx monoclonal mouse antibody (22C3, Agilent-Dako). Diaminobenzidine was used as chromogenic substrate. PD-L1 protein expression in NSCLC was determined by using the tumor proportion score (TPS), which is the percentage of viable tumor cells showing partial or complete membrane staining at any intensity. A minimum of 100 tumor cells was required to consider a sample eligible for IHC PD-L1 evaluation The scoring was reported by using a three cut-point system: score 0 (no PD-L1 expression, TPS < 1%), score 1+ (low PD-L1 expression, TPS 1–49%) and score 2 (high PD-L1 expression, TPS ≥ 50%).

### 2.3. Fluorescent In Situ Hybridization

FISH, to evaluate translocation of the *ALK* and *ROS1* genes, was performed by using the ZytoLight^®^ SPECDual Color Break Apart Probes and ZytoLight^®^ FISH-Tissue Implementation Kit (ZYTOVISION, Bremerhaven, Germania) according to the manufacturer’s instructions. The ALK and ROS1 probes included a mixture of a green fluorochrome labeled polynucleotides targeting sequences mapping in 2p23.1-p23.2 and 6q22.1, respectively and an orange fluorochrome labeled polynucleotides targeting sequences mapping to 2p23.2 in *ALK* and 6q22.1 in *ROS1*.The slides were analyzed by using a fluorescence microscope (×100). An average of 100 nuclei was considered within the invasive component of tumor tissue. A distance ≥2 signals diameter between green and orange separated signals were considered *ALK* and/or *ROS1* translocated.

Moreover, FISH was performed, as a confirmatory test, on all samples that showed an increased gene copy number variation (CNV) by NGS analysis. Gene copy-number status was assessed by using a dual-color FISH probe set (ZytolightSPEC, containing gene- and centromere-specific probes) on FFPE tissue sections from tumor specimens following established standard laboratory procedures. Gene copy number and chromosome enumeration probes (CEP) were scored in 100 cells and the mean gene/centromere ratio was calculated for each specimen.

### 2.4. Nucleic Acid Extraction and Quantification

Invasive tumor areas, identified on serial hematoxylin/eosin (H&E) sections by a pathologist, were harvested from 3 or 4 whole 5-μm paraffin sections by microdissection. DNA was isolated by using the QIAamp DNA kit (Qiagen, Milan, Italy) and RNA by using the MagCore Total RNA FFPE One-Step Kit with the MagCore Automated Nucleic Acid Extractor (RBC Bioscience, Diatech Labline, Jesi, Italy), according to the manufacturers’ instructions. DNA and RNA concentrations were determined by fluorometric quantitation by using a Qubit 2.0 Fluorimeter with Qubit DNA dsDNA BR Assay Kit and Qubit RNA BR Assay Kit (Qiagen) as appropriate.

### 2.5. Next-Generation Sequencing (NGS)

From 2016 to 2019, we used NGS to analyze 1759 patients, of which 455 were early stage, adopting the diagnostic gene panel “Oncomine Solid Tumor DNA kit (CE-IVD) (Thermo Fisher Scientific, Monza, Italy), which is able to identify more than 1900 mutations (SNV, indels) in 22 genes. Library preparation was performed on 10-ng DNA (range 1 to 20 ng) by the Ion AmpliSeq Library Kit 2.0 (Thermo Fisher Scientific) and sequencing by using the Oncomine Solid Tumor DNA kit (CE-IVD) (Thermo Fisher Scientific). The Oncomine Solid Tumor DNA kit is a single-pool panel (93 amplicons) that analyze hotspot and targeted regions of 22 genes implicated in colon and lung cancers (*KRAS*, *EGFR*, *BRAF*, *PIK3CA*, *AKT1*, *ERBB2*, *PTEN*, *NRAS*, *STK11*, *MAP2K1*, *ALK*, *DDR2*, *CTNNB1*, *MET*, *TP53*, *SMAD4*, *FBXW7*, *FGFR3*, *NOTCH1*, *ERBB4*, *FGFR1*, and *FGFR2*).

Each library was barcoded with Ion Xpress Barcode Adapters 1–16 Kit (Thermo Fisher Scientific) and diluted to a final concentration of 100 pM; barcode libraries were pooled in equimolar amounts and diluted to 35pM for downstream template preparation.). Since 2019, libraries have been constructed by using the Oncomine Focus Assay (OFA, Thermo Fisher Scientific, Inc.). The OFA panel can identify hotspot mutations, including SNVs, indels (35 genes), CNVs (Copy Number Variations, 19 genes), and 23 fusion drivers that are commonly implicated in human cancers and relevant to targeted treatment of solid tumors [19].

In particular, the DNA panel can identify hotspot mutations in the following genes: *AKT1*, *ALK*, *AR*, *BRAF*, *CDK4*, *CTNNB1*, *DDR2*, *EGFR*, *ERBB2*, *ERBB3*, *ERBB4*, *ESR1*, *FGFR2*, *FGFR3*, *GNA11*, *GNAQ*, *HRAS*, *IDH1*, *IDH2*, *JAK1*, *JAK2*, *JAK3*, *KIT*, *KRAS*, *MAP2K1*, *MAP2K2*, *MET*, *MTOR*, *NRAS*, *PDGFRA*, *PIK3CA*, *RAF1*, *RET*, *ROS1*, and *SMO*. Nineteen CNV targets are also included in the panel. Finally, the RNA assay can detect the following gene fusions: *ABL1*, *ALK*, *AKT3*, *AXL*, *BRAF*, *EGFR*, *ERBB2*, *ERG*, *ETV1*, *ETV4*, *ETV5*, *FGFR1*, *FGFR2*, *FGFR3*, *MET*, *NTRK1*, *NTRK2*, *NTRK3*, *PDGFRA*, *PPARG*, *RAF1*, *RET*, and *ROS1*. Complementary DNA (cDNA) synthesis prior to library preparation for RNA panel was carried out by using the SuperScript™ VILO™ cDNA Synthesis Kit (Thermo Fisher Scientific). Consequently, we analyzed the remaining 202 patients, of which 59 were early stage, by using this second comprehensive gene panel, and we tested 14 out of 1961 patients with both panels.

Automated library building was performed on the Ion Chef™ by using the Ion AmpliSeq™ Kit for Chef DL8 (Thermo Fisher Scientific) according to the manufacturer’s protocol. A total of 8 libraries were constructed per run. For DNA samples, library building was performed with a DNA input of 10 ng per sample (15 μL of 0.67 ng/μL dilution), with 21 amplification cycles, and annealing and extension times of 4 min. For RNA samples (cDNA), library building was performed with a cDNA input of 10 ng per sample (15 μL of 0.67 ng/μL dilution), with 31 amplification cycles, and annealing and extension time of 4 min. All libraries were labeled with IonCode Barcodes (Thermo Fisher Scientific).

Barcoded libraries were pooled and diluted to 33pmol/L in a 4:1 ratio (DNA to RNA) for downstream template preparation. Template preparation was performed with the Ion Chef system (Thermo Fisher Scientific), which integrates library amplification, ISP recovery-enrichment, and chip loading. Sequencing was performed by using the 510™ & Ion 520™ & Ion 530™ Kit on Ion GeneStudio™ S5 Prime System with Ion 520 chips. Analysis was carried out by using Ion Torrent Suite™ Software version 5.4 and Ion Reporter™ version 5.4. The Torrent Suite™ Software was used to perform initial quality control including chip loading density, median read length and number of mapped reads. The Coverage Analysis plugin was applied to all data and used to assess amplicon coverage for regions of interest. Automatic workflow with preconfigured parameter settings (Oncomine Variants 5% CI CNV ploidy ≥ gain of 2 over normal) was utilized. For data analysis of the DNA panel, a cut-off of 500x coverage was applied. Only single-nucleotide variants (SNVs) resulting in a nonsynonymous amino acid change, or a premature stop codon, and all short indels resulting in either a frameshift or insertion/deletion of amino acids were selected. All variants were manually reviewed with Integrative Genomics Viewer (IGV v.2.8.0, Broad Institute, Cambridge, MA, USA) and with the support of publicly available datasets reporting on their established or predicted oncogenicity (i.e., COSMIC, cBioPortal, Clinical Trials, ClinVar, dbSNP, dbVar, Catalog of somatic mutations in cancer, My Cancer genome, personalized cancer therapy, NCBI genome, RefSeqGene and Locus reference Genomic). The detection of a rearrangement in a sample was judged as true positive following the manufacturer’s recommendations (Thermo Fisher Scientific). Visualization of detected fusion events was made by using Integrative Genomics Viewer (IGV; Broad Institute, Cambridge, MA, USA) demonstrating the alignment of sequenced reads to the reads of known fusion breakpoints and the reference human genome hg19. For further examination of NGS data, in order to prioritize variants and find the relevant cancer drivers, we used the genomic analysis software “Oncomine Reporter”.

### 2.6. Statistical Analysis

Descriptive statistics were used for all variables of interest. Continuous variables were reported as means and relative standard deviations (SD) or as medians and relative ranges, whereas categorical variables were presented through frequencies and percentage values. Differences between variables were evaluated by using the Pearson’s Chi-square test, Fisher exact test, or Mann–Whitney test, as appropriate. Overall survival (OS) analyses were carried out by using the Kaplan–Meier product-limit method in order to explore the potential prognostic role of specific mutation profiles in a subgroup of early-stage patients. OS was defined as the time lapse occurring from surgery to death or to the last follow-up, if censored. The log-rank test was used to prove whether or not there was any difference between subgroups. A *p*-value < 0.05 was considered statistically significant. All statistical analyses were conducted with SPSS v.21.0.

## 3. Results

### 3.1. Stage-Specific Analysis of NSCLC Actionable Alterations in a Single-Institution Cohort

Different panels were used for the diagnostic NGS analyses of 1961 consecutive samples of LUAD collected from January 2016 to March 2021 at the Regina Elena National Cancer Institute: 1745 patients were subjected to NGS analysis by using the Oncomine Solid Tumor 22 gene panel, 202 patients were tested by Oncomine Focus Assay 52 gene panel and 14 with both panels. (Table 1). Among the 1961 samples, 513 and 1448 were obtained from stages I–IIIA (collectively referred to as early-stage) and stage IIIB–IV (collectively referred to as advanced-stage) tumors. Median age at diagnosis was 69 (range 23–90) years; male and female patients were 58% and 42%, respectively (Table 1). Overall, the most frequently mutated genes (Table 1) were *KRAS* (27.3%), *TP53* (25.8%), *EGFR* (13.8%), *MET* (exon-skipping mutations, 6.3%), *BRAF* (3.8%), *PIK3CA* (3.8%), and *ERBB2* (0.9%). *ALK*, *RET*, and *ROS1* fusion genes were detected in 5.5%, 3.4%, and 0.5% of cases, respectively. We detected only one case that was positive for *NTRK1* fusion. These percentages agree substantially with previously reported data on the NSCLC mutational landscape [20,21] Anti-PD-L1 immunoreactivity was detected in 42.0% (score 0), 37.8% (score 1), and 20.2% (score 2) of cases (Table 1).

Comparing early- versus advanced-stage cases (Table 2), patients were found to have a younger age at diagnosis in the advanced-stage group (*p* = 0.023). Although we cannot offer a clear-cut explanation for this finding, we suggest that there could be a bias toward dismissing the likelihood of lung cancer diagnosis in younger patients, consequently increasing the frequency of diagnosis in the advanced stage [22]. We did not observe any significant differences in the overall prevalence of *EGFR* mutations when tumors from early- (13.6%) and advanced- (13.8%) stage patients were compared (Table 2). This was paralleled by a similar prevalence of other genomic alterations reported in Table 2, except that *KRAS* mutations were more frequent in early-stage cases (*p* = 0.045). Of clinical significance, the prevalence of the actionable *KRAS* p.G12C mutation was comparable in the two groups (10.3% in early and 10.1% in late stage, respectively).

**Figure 1:**
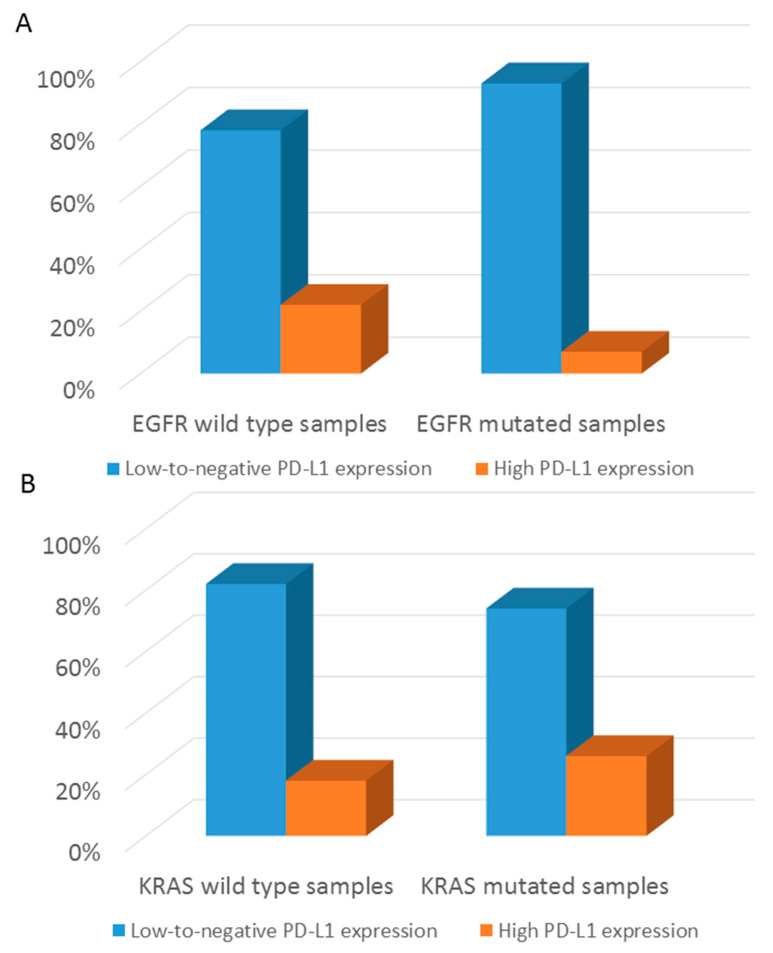

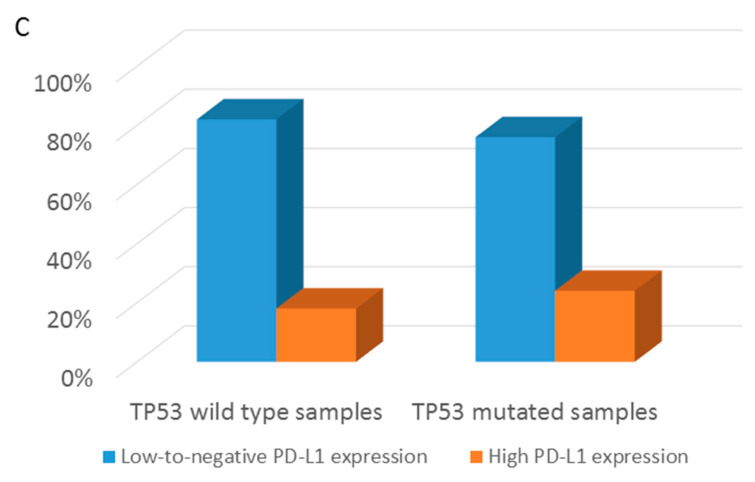
Associations between PD-L1 expression and genomic alterations in the entire cohort. (**A**) Bar graphs show the percentage of *EGFR*-mutated samples with either low-to-negative PD-L1 expression or high PD-L1 expression. (**B**) Bar graphs show the percentage of *KRAS*-mutated samples with either low-to-negative PD-L1 expression or high PD-L1 expression. (**C**) Bar graphs show the percentage of *TP53*-mutated samples with either low-to-negative PD-L1 expression or high PD-L1 expression.

Score 1 anti-PD-L1 immunoreactivity was comparable between early and advanced stages, whereas score 0 and score 2 PD-L1 expression trended toward higher representation in early and advanced stages, respectively, without reaching statistical significance (note that PD-L1 evaluation was performed prior to radiotherapy/chemotherapy in all the early and advanced patients included in this study). Unlike the results published by Evans et al. [23], we found no significant difference in PD-L1 expression comparing surgical resection versus biopsy samples (Table S1), primary tumors versus metastases (Table S2), and locally advanced tumors (stages IIIA and IIIB) versus metastatic tumors (stage IV) (Table S3). Focusing on associations between PD-L1 expression and genomic alterations, early- and advanced-stage tumors showed an inverse association between *EGFR* mutations and high (score 2) PD-L1 expression (*p* < 0.001) (Figure 1A) and a positive association between *KRAS* genomic alterations (all variants, including p.G12C) and score 2 PD-L1 reactivity (*p* = 0.001) (Figure 1B). Moreover, *TP53* mutated tumors were more frequently characterized by high PD-L1 expression (Figure 1C).

**Figure 2:**
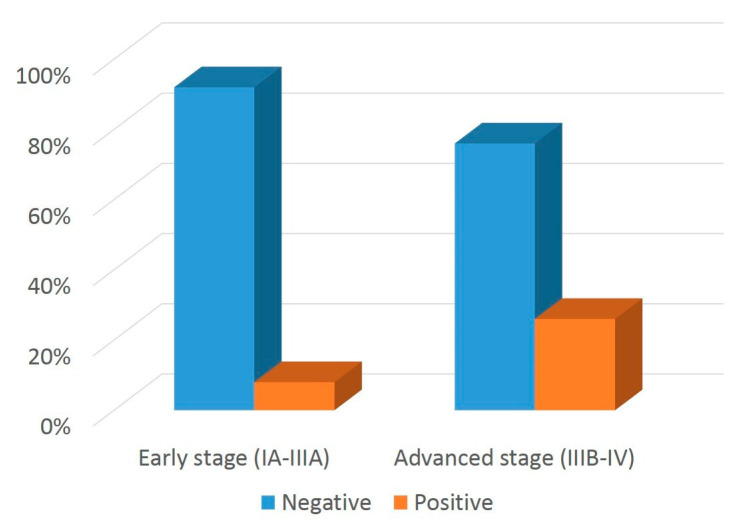
CNV (copy number variation) distribution in early- and advanced-stage tumors. Bar graphs show the percentage of samples that scored positive or negative for CNV detection.

Copy number gains were investigated with the Oncomine Focus panel. Of 216 cases available for analysis, 166 reached the ≥50% cut-off value for neoplastic cell content and could be evaluated. Of those 166 cases, 59 were early- and 107 advanced-stage. As shown in Figure 2, copy number gains were significantly higher (*p* = 0.012) in advanced-stage tumors (24%) compared to early-stage lesions (8%). The most frequently altered genes were *MYC* (16%), *EGFR* (13%), *MET* (13%), *CDK4* (10%), *KRAS* (10%), *CCND1* (6%), and *PIK3CA* (6%). All the copy number gains were confirmed by FISH analysis.

### 3.2. Distribution of EGFR Mutation Classes in Early Stage NSCLC

**Figure 3:**
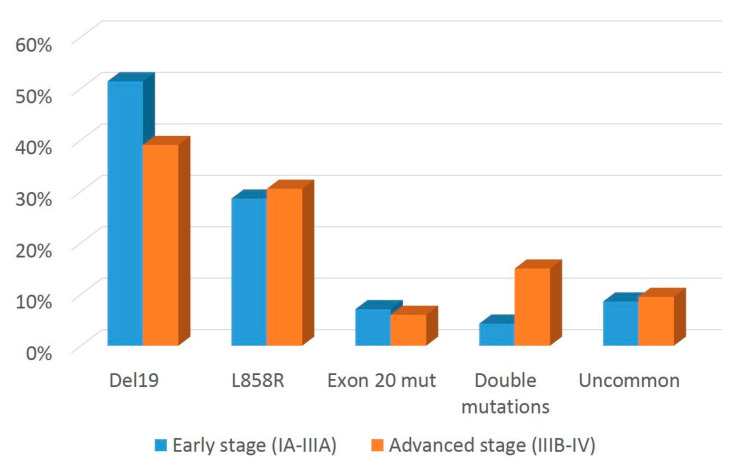
*EGFR* genomic alterations in early- and advanced-stage tumors. Bar graphs show the percentage distribution of distinct subgroups of *EGFR* mutations in early- versus advanced-stage LUAD patients.

Adjuvant osimertinib was approved by the FDA for the p.L858R variant and mutations generated by exon 19 deletion, i.e., the most prevalent *EGFR* genomic alterations, although, in principle, patients carrying other low-prevalence *EGFR* mutations might benefit from osimertinib treatment [24,25]. As shown in Figure 3, *EGFR* genomic alterations for which adjuvant osimertinib is not currently approved were grouped as exon 20 mutations, double mutations—i.e., two different mutations, not including p.T790M, located in two different *EGFR* exons—and uncommon mutations, which included all the remaining genomic alterations not falling within the above categories. Note that three primary pT790M mutations (one in early-stage and two in advanced-stage cases) were enlisted among exon 20 mutations.

Exon 19 deletions tended to be more frequent in early versus advanced stage (51.4% and 39%, respectively), although this difference did not reach statistical significance (*p* = 0.137). Double mutations were more frequent in advanced versus early (15% vs. 4.3%) stage tumors, a datum that must be considered with caution given the low number of early-stage samples harboring this class of *EGFR* genomic alterations. Notably, in our series, non-exon 19 deletions and non-L858R mutations predicted to be sensitive to osimertinib [26] (i.e., uncommon and double mutations) represented 13% of *EGFR* genomic alterations in stage IB-IIIA cases (exon 20 mutations, excluding T790M, are resistant to osimertinib).

**Figure 4:**
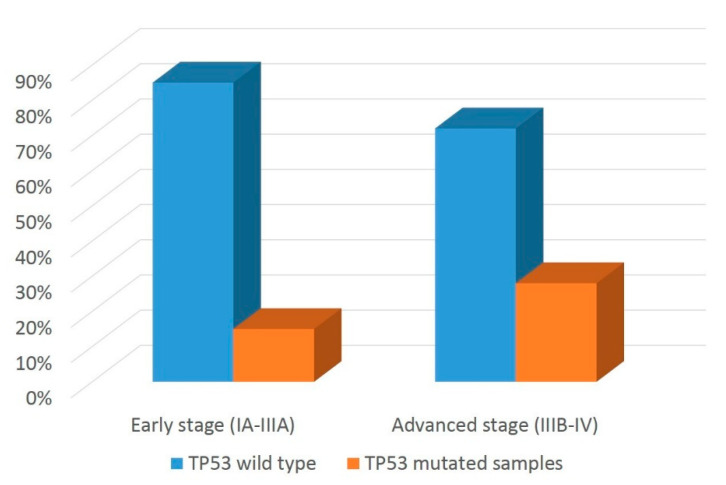
*TP53* status in *EGFR* mutated cases in early- and advanced-stage tumors. Bar graphs show the percentage of *EGFR*-mutated samples with either wild-type or mutated *TP53* genotype.

Given that co-occurrence of *EGFR* and *TP53* mutations has a negative impact on clinical responses to EGFR inhibitors [27], we investigated the fraction of early- and advanced-stage tumors in which *EGFR* and *TP53* GAs co-occurred. Results from this analysis are shown in Figure 4 and indicate that *TP53* mutations co-occurred with *EGFR* variants at a significantly higher rate in advanced vs. early-stage tumors (28% versus 15%, *p* = 0.022).

### 3.3. Distribution of Targetable Alterations in Early-Stage NSCLC Based on Histological Pattern

**Figure 5:**
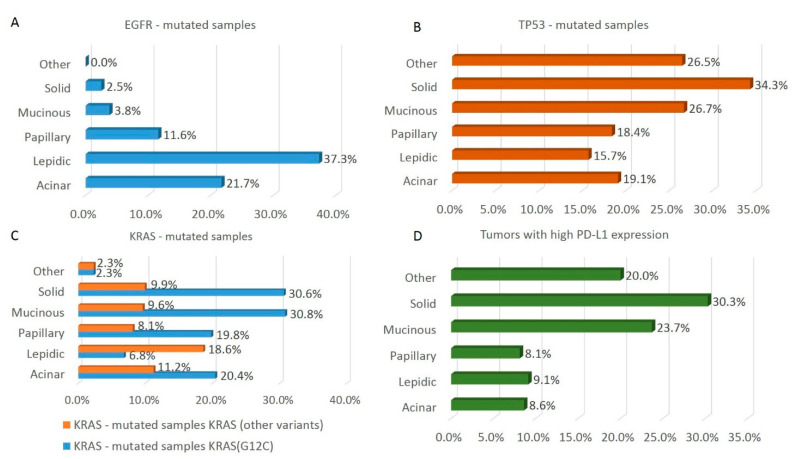
Distribution of histological patterns in *EGFR-*, *TP53-* or *KRAS*-mutated samples and in high PD-L1 expression tumors. (**A**) The percentage of the indicated LUAD histology patterns in *EGFR*-mutated samples is reported. (**B**) The percentage of the indicated LUAD histology patterns in *TP53*-mutated samples is reported. (**C**) The percentage of the indicated LUAD histology patterns in G12C (orange bars) and non-G12C (blue bars) *KRAS*-mutated samples is reported. (**D**) The percentage of the indicated LUAD histology patterns in tumors with high PD-L1 expression is reported.

To further dissect the genomic profile of early-stage NSCLC, we grouped tumors according to their histological pattern and found that the acinar, solid, papillary, lepidic, and mucinous subtypes accounted for 29.6%, 23.6%, 16.8%, 11.5%, and 10.1% of the total, respectively. The remaining 8.4% of cases contained minor histotypes, which were grouped together in a single pool. In agreement with data reported on the overall population—i.e., stage-agnostic prevalence of *EGFR* mutations in NSCLC—we found that the lepidic (37.3%) and acinar (21.7%) subtypes were those most enriched for *EGFR* mutations also in early-stage NSCLC, whereas the solid and mucinous subtypes harbored *EGFR* mutations in only 2.5% and 3.8% of cases (Figure 5A). Although *EGFR* rarely mutated, the mucinous and solid histological subtypes harbored *TP53* mutations most frequently (26.7% and 33.4%, respectively, Figure 5B). In contrast with the data on *EGFR* variants, *KRAS* mutations were mostly distributed among mucinous and solid histotypes (40% each). Interestingly, however, the lepidic subtype contained the highest proportion, relative to all *KRAS* mutations, of the actionable *KRAS* p.G12C variant [28] (Figure 5C). Finally, anti-PD-L1 score 2 immunoreactivity was over-represented in solid NSCLC samples, (30.3%, Figure 5D).

### 3.4. Overall Survival Analysis

Data which was considered suitable to carry out overall survival (OS) analyses were available for a subgroup of patients (*n* = 223) who underwent surgery and received no further treatment (including chemotherapy), because of clinical staging and/or comorbidity assessment. Median follow-up was 30 months range (1–65) months.

**Figure 6:**
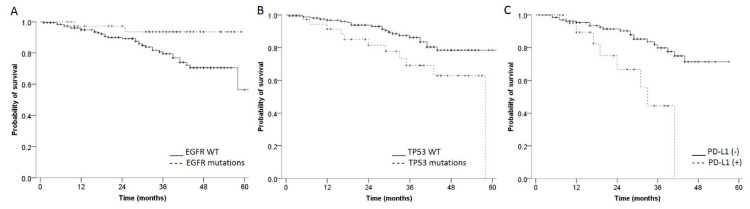
Overall survival plots according to *EGFR*, *TP53* mutational status and PD-L1 expression. Kaplan–Meier curves show differences in overall survival according to *EGFR* (**A**) or *TP53* (**B**) mutational status. A similar analysis was performed in panel (**C**) by separating tumors with high (+) and low-to-negative PD-L1 expression (-) (see Materials and Methods for details).

The survival rate at 3- and 5-years post-surgery was 82.2% and 62.2%, respectively. Statistically significant differences were observed between patients’ subgroups, which differed in their *EGFR* and *TP53* mutation status. In particular, *EGFR*-mutated patients had longer survival compared to those with wild-type *EGFR* status (survival rate at 3 years: 93.5% vs. 79.5% *p* = 0.039) (Figure 6A), whereas patients with mutated *TP53* had shorter survival in comparison to the wild-type *TP53* subgroup (survival rate at 3 years: 69.1% vs. 86.2% (*p* = 0.018) (Figure 6B). Focusing on PD-L1 (Figure 6C), patients with high PD-L1 expression had a shorter survival rate compared to those with low-to-negative PD-L1 expression (survival rate at 3 years: 44.5% vs. 81.8%, *p* = 0.001). No differences in overall survival were observed in patients with any of the other molecular alterations investigated in this study (data not shown).

## 4. Discussion

Genotype-matched therapeutics have been used for the treatment of locally advanced and metastatic NSCLC for more than a decade. Thus, clinical and translational studies on NSCLC-targeted therapies have focused mostly on this clinical setting. The recent approval of osimertinib as adjuvant monotherapy for *EGFR*-mutated stage IB-IIIA NSCLC suggests that, in the near future, other NSCLC driver mutations could be targeted in the adjuvant setting. This scenario calls for studies aimed to further our knowledge about the prevalence of mutations affecting actionable genomic drivers in the routine diagnosis of early-stage NSCLC.

Since 2016, in our institution, NSCLC samples have been screened for actionable genomic alterations and the most prevalent driver mutations, irrespective of disease stage. This allowed us to assess the spectrum of actionable genomic alterations in general—and *EGFR* genomic alterations in particular—in 1961 consecutive NSCLC cases, 513 (26.2%) and 1448 (73.8%) of which were classified as early and advanced stage, respectively. In our series, currently actionable genomic alterations had a similar prevalence in early and advanced NSCLC, showing a substantial overlap with previously reported data on the NSCLC mutational landscape in patients of European descent [20,21]. Therefore, our data show that targeted NGS analyses that use relatively small gene panels provide reliable data, suitable for the clinical management of early-stage NSCLC cases.

From the biological standpoint, it is reassuring that we obtained genetic evidence of tumor progression, as exemplified by the increased CNV frequency in advanced versus early-stage NSCLC. Moreover, we found that *EGFR* and *TP53* were co-mutated with significantly higher frequency in advanced cases. These observations are in line with the concept that tumor progression is driven by the sequential accumulation of evolutionarily advantageous genomic alterations.

Concerning potential correlations between genomic analyses and overall survival of early-stage untreated patients, we observed that wild-type *EGFR* status was associated with worse overall survival. Previous studies investigating the association of *EGFR* mutations with survival outcomes in patients with resected NSCLC have yielded conflicting results [29]. It is possible that the association of *EGFR* mutations with favorable prognostic factors might act as a confounding factor in these types of analysis. Furthermore, given the large spectrum of *EGFR* mutations in LUAD, it remains to be seen whether prognosis is affected differently by distinct mutations or mutation subgroups [30]. *TP53* status was also informative: thus, in line with previous observations [31], our study found that *TP53* mutations had negative prognostic value in early-stage LUAD patients. It therefore appears that *TP53* status is potentially useful for clinical decisions regarding the post-surgery management of LUAD patients.

Concerning PD-L1, our data highlighted that a sizeable fraction of early-stage NSCLC (16.5%, see Table 2) expressed PD-L1 within the threshold values currently used to assign patients with advanced-stage NSCLC to therapy with immune checkpoint inhibitors. Moreover, high PD-L1 expression in early-stage LUAD patients was associated with worse overall survival, in agreement with a meta-analysis which found that PD-L1 positivity was inversely associated with survival after surgical resection in early-stage LUAD [32]. Therefore, besides its emerging role as a predictive biomarker of response to immune checkpoint inhibitor-based adjuvant immunotherapy, PD-L1 expression in cancer cells also appears to be a solid candidate prognostic biomarker for early-stage LUAD patients.

Concerning our more detailed studies on *EGFR* mutations, these were found to be distributed unevenly across different histological subtypes in early-stage NSCLC, which in fact reflected the pattern observed in advanced NSCLC. Accordingly, diagnosis of acinar- or lepidic-histological pattern must be taken as one of the possible characteristics that could indicate the presence of actionable *EGFR* mutations in early-stage NSCLC. We found that about 12% of *EGFR* variants in our series (i.e., double mutations and uncommon mutations) did not fall within the perimeter of *EGFR* genomic alterations for which adjuvant osimertinib gained FDA and EMA approval, despite being predictive of sensitivity to osimertinib based on data consolidated in advanced-stage LUAD. This issue deserves greater attention, if the benefit of adjuvant osimertinib is to be extended to as many patients with early-stage NSCLC as possible. Of potential clinical relevance is our finding that 14% of *EGFR* mutations in early-stage NSCLC co-occurred with *TP53* mutations. In light of the consolidated notion that *TP53* genomic alterations have a negative impact on therapeutic responses to *EGFR* tyrosine kinase inhibitors in advanced *EGFR*-mutated NSCLC [27,33], it will be interesting to assess whether *TP53* mutations have an impact on responses to osimertinib also in early-stage NSCLC patients.

Based on the paradigm of *EGFR* targeting by osimertinib, it is becoming clear that other genotype-matched therapeutics, currently approved for advanced-stage NSCLC, might provide clinical benefit in the adjuvant setting. This hypothesis is currently being tested in a Phase 2 clinical trial (NCT04302025, www.clinicaltrials.gov, first posted 10 march 2020), which is evaluating responses to genotype-matched drugs in resectable NSCLC that carry *ALK*, *ROS1*, *NTRK*, *BRAF*, and *RET* driver mutations.

## 5. Conclusions

As a final consideration, we would like to point out that our data reflect a real-world setting, which inevitably skews representation in favor of advanced cases. Despite this caveat, we believe that our sample size is sufficiently high to draw a reliable and clinically valuable picture. In this respect, we note that the number of clinically actionable mutations in LUAD is expected to increase in the near future, as novel drugs currently in clinical development eventually obtain regulatory approval. Within this framework, it will be important to carry out studies with larger cohorts of patients. By keeping an eye on differences based on patients’ ethnicity, this approach will make it possible to obtain a better estimate of the prevalence of less common actionable drivers, including oncogenic fusions. Hence, we believe that our data provide the basis for larger confirmatory studies of genomic alterations that are predictive of sensitivity/resistance to genotype-matched therapeutics.

## Figures and Tables

**Table 1:** Patient characteristics.

Variable	*N =* 1961	%
Gender		
Female	826	42%
Male	1135	58%
Age at molecular diagnosis		
Median (min–max)	69 (23–90)	
Stage		
Early (IA–IIIA)	513	26%
Advanced (IIIB–IV)	1448	74%
Type of panel		
22 Genes	1745	89%
Focus	202	10%
Both	14	1%
*EGFR*	270/1961	13.8%
*ALK*	107/1951	5.5%
*ROS1*	9/1956	0.5%
*BRAF*	71/1890	3.8%
V600E	24/1890	1.3%
Other variant	47/1890	2.7%
*KRAS*	536/1961	27.3%
G12C	199/1961	10.1%
Other variant	337/1961	17.2%
*MET* exon 14 skipping	13/207	6.3%
*RET* fusions	7/207	3.4%
*TP53*	454/1759	25.8%
*PIK3CA*	75/1961	3.8%
*ERBB2*	17/1961	0.9%
PD-L1		
score 0 (<1%)	568/1351	42.0%
score 1 (1–49%)	512/1351	37.8%
score 2 (≥50%)	271/1351	20.2%

**Table 2:** Patients’ characteristics according to clinical stage.

Variable	Early Stage	Advanced Stage	*p*-Value
	*N* (%)	*N* (%)	
	513	1448	
Gender			0.078
Female	233 (45%)	593 (41%)	
Male	280 (55%)	855 (59%)	
Age at molecular diagnosis			0.023
Median (min–max)	70 (27–86)	68 (23–90)	
*EGFR*	70/513 (13.6%)	200/1448 (13.8%)	0.925
*ALK*	25/513 (4.8%)	82/1438 (5.7%)	0.479
*ROS1*	2/513 (0.39%)	7/1443 (0.48%)	0.784
*BRAF*	18/513 (3.5%)	53/1448 (3.6%)	0.543
V600E	10 (1.9%)	37 (2.5%)	
Other variant	8 (1.5%)	16 (1.1%)	
*KRAS*	159/513 (30.9%)	377/1448 (26%)	0.045
G12C	53/513 (10.3%)	146/1448 (10.1%)	
Other variant	106/513 (20.6%)	231/1448 (15.9%)	
*MET* exon 14 skipping	3/58 (5.2%)	10/149 (6.7%)	0.682
*RET*	2/58 (3.4%)	5/149 (3.3%)	0.974
*TP53*	107/455 (23.5%)	347/1304 (26.6%)	0.194
*PIK3CA*	16/513 (3.1%)	59/1448 (4.1%)	0.332
*ERBB2*	4/513 (0.8%)	13/1448 (0.9%)	1.000
PD-L1			
score 0 (<1%)	163/357(45.7%)	405/994 (40.7%)	0.104
score 1 (1–49%)	135/357 (37.8%)	377/994 (37.9%)	
score 2 (≥50%)	59/357 (16.5%)	212/994 (21.4%)	

## Data Availability

The datasets used and/or analyzed during the current study are available from the corresponding author on request.

## Supplementary Materials

The following supporting information can be downloaded at: https://www.mdpi.com/article/10.3390/cancers14122971/s1, Table S1: Relationship between PD-L1 expression and sampling modality; Table S2: Relationship between PD-L1 expression and tissue type; Table S3: Relationship between PD-L1 expression and advanced or metastatic stages.
