# Supplementary material for: A Real-World Systematic Analysis of Driver Mutations’ Prevalence in Early- and Advanced-Stage NSCLC: Implications for Targeted Therapies in the Adjuvant Setting
Source: Cancers (Basel). 2022 Jun 16;14(12):2971. doi: 10.3390/cancers14122971 (PMC9221477; doi:10.3390/cancers14122971)
Supplement: Supplementary file 1 [file cancers-14-02971-s001.zip › cancers-1651885-supplementary.pdf]

Table S1. Relationship between PD-L1 expression and sampling modality.

PD-L1	Type of sample		Total
	Surgical sample	Cytology or Biopsy	
	N (%)	*N* (%)	*N* (%)
**score 0–1**	364 (34)	716 (66)	1080 (100)
**score 2**	77 (28)	194 (72)	271 (100)
**Total**	441 (33)	910 (67)	1351 (100)

The table shows no significant difference in PD-L1 expression comparing surgical resection vs. biopsy or cytology samples ($p = 0.097$).

Table S2. Relationship between PD-L1 expression and tissue type.

PD-L1	Type of diagnosis		Total
	Primary	Metastatic	
	N (%)	*N* (%)	*N* (%)
**score 0–1**	698 (65)	382 (35)	1080 (100)
**score 2**	160 (59)	111 (41)	271 (100)
**Total**	858 (64)	493 (36)	1351 (100)

The table shows no significant difference in PD-L1 expression comparing primary tumors vs. metastases ($p = 0.087$).

Table S3. Relationship between PD-L1 expression and advanced or metastatic stages.

PD-L1	Advanced or Metastatic stages		Total
	stage IIIA+IIIB	stage IV	
	N (%)	*N* (%)	*N* (%)
**score 0–1**	215 (25)	637 (75)	852 (100)
**score 2**	59 (27)	162 (73)	221 (100)
**Total**	274 (26)	799 (74)	1073 (100)

The table shows no significant difference in PD-L1 expression comparing locally advanced tumors (stages IIIA and IIIB) *versus* metastatic tumors (stage IV) ($p = 0.657$).
